# Enhanced Mixing in Microflow Systems Using Magnetic Fields—Experimental and Numerical Analyses

**DOI:** 10.3390/mi16040422

**Published:** 2025-03-31

**Authors:** Marek Wojnicki, Xuegeng Yang, Piotr Zabinski, Gerd Mutschke

**Affiliations:** 1 Faculty of Non-Ferrous Metals, AGH University of Science and Technology, Mickiewicza Ave. 30, 30-059 Krakow, Poland; zabinski@agh.edu.pl; 2 Institute of Fluid Dynamics, Helmholtz-Zentrum Dresden-Rossendorf, 01328 Dresden, Germany; x.yang@hzdr.de

**Keywords:** microflow systems, magnetic field mixing, enhanced mixing, passive mixing, active mixing, magnetic field-driven mixing

## Abstract

This study presents both numerical and experimental analyses of enhanced mixing in a microflow system under the influence of a magnetic field. The research employed COMSOL Multiphysics for numerical simulations and Particle Image Velocimetry (PIV) for experimental validation. In the experimental microfluidic setup, permanent neodymium magnets were used to influence a laminar flow of water partially enriched with Ho(III) ions using the magnetic field. The findings confirmed that the strong interaction between Ho(III) ions and the magnetic field significantly affected the flow and may have resulted in vortex shedding downstream of the region with the highest magnetic field intensity. The numerical simulations demonstrated good agreement with the PIV experimental results. These findings suggest that it is possible to significantly enhance mixing in microflow systems without mechanical components, solely by exploiting the differences in the magnetic properties between the mixing substances. Traditionally, microreactors have been limited by mixing speeds governed by diffusion. These new results indicate the practical possibility of increasing mixing intensity in a cost-effective and safe manner.

## 1. Introduction

The concept of microflow reactors, also known as microreactors, emerged in the late 20th century as part of the broader development of microfluidics and lab-on-a-chip technologies. These systems offer a range of advantages over traditional large-scale reactors, including better control over reaction conditions, enhanced safety due to smaller volumes of reactive materials, and the ability to handle hazardous or exothermic reactions more safely. Additionally, the high surface-area-to-volume ratio in microreactors allows for efficient heat transfer, making them ideal for both endothermic and exothermic reactions [1,2,3].

The data presented in the bar chart were extracted from the Scopus database. A comprehensive search was conducted using the following query: “microflow AND system AND mixing”. The search criteria included “all fields,” meaning that titles, abstracts, keywords, and other relevant metadata were analyzed to gather comprehensive results.

**Figure 1:**
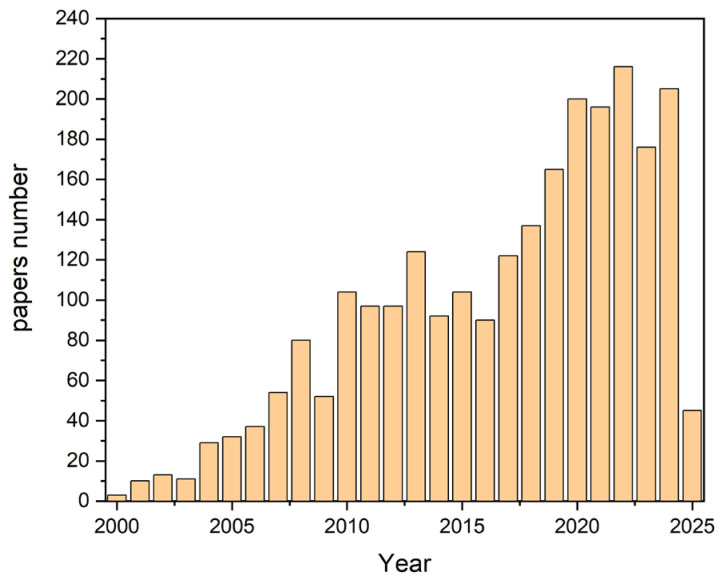
Number of publications on microflow system mixing over time based on Scopus database search results.

The bar chart clearly illustrates a strong upward trend in the number of publications over the years, highlighting the increasing interest and growth in research related to mixing in microreactors (see Figure 1).

This trend reflects the expanding significance of microflow systems in scientific research and industrial applications. The growing number of studies indicates an increased focus on improving mixing efficiency, exploring new methodologies, and enhancing microreactor designs to meet the demands of modern scientific advancements.

Despite these benefits, microreactors also face certain challenges. One of the key limitations is the difficulty of mixing fluids at the microscale [4]. In such small volumes, the flow is typically laminar, meaning that fluids flow in parallel layers with minimal cross-mixing. This laminar nature is due to the dominance of viscous forces over inertial forces at small scales, which contrasts with the turbulent flow that typically promotes mixing in larger systems. Consequently, mixing in microreactors often relies solely on diffusion, a slow process that can limit the efficiency of chemical reactions, particularly when rapid mixing is essential [5]. To overcome this challenge, researchers have developed various strategies for enhancing mixing in microreactors, which are generally divided into two categories: passive and active mixing. Passive mixing relies on the geometry of the microchannels to induce chaotic advection or increase the contact surface between fluids. Examples include zigzag channels [6], obstacles, and helical structures [7,8]. Staggered non-abreast baffles (SNBs) and staggered abreast baffles (SABs) can significantly enhance mixing by creating chaotic convection, especially at low Reynolds numbers (Re) [9]. Sharp corner structures in microchannels can induce three-dimensional vortices, stretching and folding fluid interfaces to increase the interfacial contact area and improve mixing efficiency [10,11].

While passive mixing requires no external energy input, its effectiveness is often limited by the Reynolds number, which remains low in microflow systems.

In contrast, active mixing uses external forces or energy sources to enhance fluid mixing. Techniques such as ultrasonic waves [12], electrical fields [13], magnetic fields [14], and temperature gradients [15] are employed to disturb the laminar flow and induce vortices or other flow disturbances that promote mixing. Active mixing methods can achieve faster mixing, but they often involve more complex designs, higher costs, and increased energy consumption. Active mixing methods, such as those involving time-pulsing flow, electrical fields, and ultrasound, can significantly improve mixing efficiency by creating more dynamic and controllable mixing environments [16]. Active mixing methods often require substantial energy input, making them less energy-efficient than passive methods. For instance, the use of electrical fields or ultrasound can be energy-intensive [17]. The design and implementation of active mixing systems can be complex and costly. The reasons include the need for specialized equipment and maintenance, which can increase overall operational costs [18].

The last decade has seen significant advancements in microfluidic mixing methods, with a focus on achieving efficient mixing in microscale devices through techniques such as chaotic advection, surface acoustic waves, and vibration-induced flow. However, challenges related to low Reynolds numbers, optimization of micromixer geometries, and mixing in stagnant fluids persist, highlighting the need for further research in this area. Table 1 presents selected mixing methods and their advances and challenges.

Magnetic mixing typically involves the use of magnetic beads or particles that are manipulated by external magnetic fields. These particles can create secondary vortices and chaotic motion within the microchannels, enhancing mixing [26,27,28]. When magnetic nonspherical particles (MNSPs) rotate under an external magnetic field, they generate secondary vortices that expand the mixing zone. The presence of multiple MNSPs can create a chaotic vortex area, further improving mixing efficiency [26].

Magnetic mixing can achieve high mixing efficiencies, particularly in low Reynolds number flows. The rotation and movement of magnetic particles under a magnetic field can create localized vortices that enhance mixing [26,27,28].

Both chaotic mixing and magnetic mixing methods are effective in creating localized vortices and enhancing mixing efficiency in microfluidic systems. Chaotic mixing relies on the manipulation of fluid flow patterns to achieve chaotic advection, while magnetic mixing uses the movement of magnetic particles to generate vortices. Each method has its advantages and specific applications, with chaotic mixing being more versatile in terms of design and magnetic mixing offering high efficiency with the use of external magnetic fields [29,30].

Our study introduces a novel approach to active mixing in microreactors, leveraging differences in the magnetic susceptibility of various substances to induce mixing [31]. Unlike conventional methods that utilize magnetic nanoparticles or microparticles, our approach relies solely on the inherent magnetic properties of the mixed substances. By applying a magnetic field, we create disturbances in the flow that promote enhanced mixing without requiring mechanical components or complex channel geometries. The effectiveness of this method is directly related to the magnitude of the magnetic susceptibility difference between the mixed substances—the greater the difference, the more efficient the mixing [32]. To illustrate this concept, we suggest incorporating a table that presents the magnetic susceptibility values of various water-soluble substances, such as holmium salts, iron salts, copper salts, selected organic compounds, and water itself (see Table 2). This comparison would further emphasize the significance of magnetic susceptibility differences in achieving effective mixing in microreactors.

In our experiments, we used neodymium magnets to generate a strong magnetic field, and Ho(III) ions were introduced into the fluid to take advantage of their paramagnetic properties. We investigated how the magnetic field influenced the flow, particularly in terms of vortex formation, and validated our findings through numerical simulations conducted with COMSOL Multiphysics. These simulations allowed us to assess the impact of magnetic field strength and ion concentration on flow behavior and mixing efficiency. These efforts were stimulated through initial 2D simulations, which demonstrated that superior mixing was achieved downstream of the channel position where permanent magnets were positioned on the upper side.

As mentioned earlier, the use of active mixing generally requires an external energy source. However, in the case of employing a magnetic field, the reactor design remains relatively simple. Permanent magnets do not require any external energy input, making this approach not only efficient but also cost-effective and maintenance-free. This advantage makes magnetic field-driven mixing an attractive option, as it combines the benefits of active mixing with the simplicity and energy independence of passive systems.

In the context of micromixing, the use of magnetic particles [33], including ferrofluids [34], is becoming increasingly popular [35]. These techniques allow for the precise control of the mixing process at the micro level, which is crucial in areas such as microfluidics and chemical engineering [14]. The application of an external magnetic field enables control over the movement and distribution of magnetic particles, leading to effective mixing even in very small volumes [36].

Various magnetization models are used to describe the behavior of magnetic particles in these systems [33]. These models take into account aspects such as particle size, shape, material properties, and interactions between them. This makes it possible to predict and optimize the behavior of ferrofluids in specific micromixing applications [14].

Prospects for further development in this field include the integration of ferrofluids with modern microfluidic technologies, which could lead to the creation of more advanced and precise mixing systems [27]. Additionally, research into new magnetic materials and a better understanding of magnetization mechanisms may contribute to the development of more efficient and versatile ferrofluids for micromixing applications.

Our work provides insights into the potential for magnetic fields to drive mixing in microflow systems, offering a cost-effective and energy-efficient solution to one of the major challenges in microreactor technology. This approach opens new possibilities for optimizing microreactor designs, particularly in applications where fast and efficient mixing is crucial.

## 2. Materials and Methods

The model flow system was designed using SolidWorks software (version 2019). The designed model was then 3D printed using a FormLabs 3D printer (Formlabs, Krakow, Poland, model form 3) with transparent resin. After printing, the component was washed with isopropanol (analytical grade), followed by curing under UV radiation. To achieve optical transparency of the surfaces, they were polished first with sandpaper (2000 grit) and then with polishing paste.

To pump the solutions, syringe pumps (Harvard Apparatus) were used. This study utilized aqueous solutions, including hydrochloric acid (HCl) from Warchem, A.P. and HoCl_3_ from Onyxmet, Poland (analytical grade purity). HoCl_3_ salts are colored, allowing for spectrophotometric analysis.

To track the flow, 10 µm of fluorescent microparticles from microParticles GmbH were used. The PIV setup used in this study included a laser (Photonics, New York, NY, USA, Nd:YLF 527 nm, 20 mJ) and a high-speed camera (VEO 410L, Phantom, Wayne, NJ, USA), both connected to a stereo microscope (ZEISS, Oberkochen, Germany, SteREO Discovery.V8). The UV–vis spectra were obtained and the kinetic measurements were performed using a UV-2700 spectrometer (Shimadzu, Kyoto, Japan).

**Figure 2:**
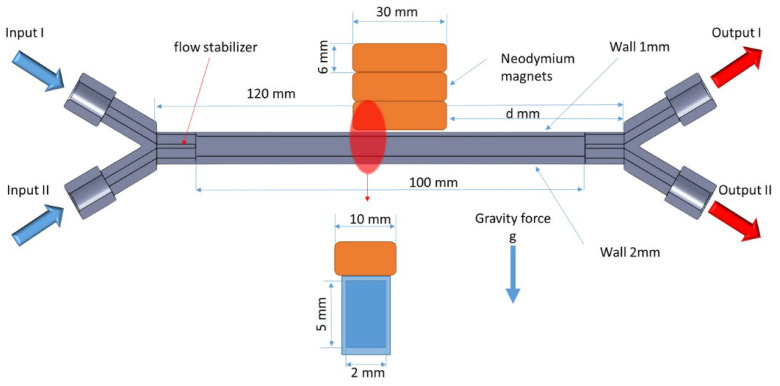
Schematic diagram of the setup for analyzing the influence of a magnetic field on the flow of liquids.

Figure 2 presents a schematic of the experimental setup used to investigate the influence of a magnetic field on fluid flow. The system was equipped with two input channels (Input I and Input II) and two output channels (Output I and Output II). In a typical experiment, Input I was supplied with deionized water, while Input 2 was supplied with an acidic solution containing 0.1 M HoCl_3_. Under laminar flow conditions, a sharp boundary between the colored solution (containing Ho(III)) and the deionized water was visible.

The Output I and Output II channels were connected to PTFE tubing with a diameter of 500 μm, and the samples were collected into test tubes. The output system was designed in such a way that the pressure differences resulting from the height variations of the tubes were compensated for. This ensured that the flow rates from Output I and Output II were equal. As a result, in a typical experiment, Output I consisted of approximately 98% pure water, while Output II consisted of approximately 98% Ho(III) solution. Concentration fluctuations at the outputs mainly resulted from imperfections in the pressure compensation system.

Numerical simulations of the flow experiments were conducted using COMSOL Multiphysics software V. 5.5.

A single cuboid NdFeB magnet with dimensions of 6 × 10 × 30 mm was used to generate a magnetic field of 0.3016 T at the center of its large surface. Piling up a number of magnets allowed the magnetic field strength to be increased, as shown below in Table 3.

As can be seen, assembling more than six magnets only moderately increased the magnetic field due to the growing distance of the upper magnets.

## 3. Results

Upon applying a magnetic field, the Ho(III)-rich phase in the microflow channel was attracted to the surface of the magnet and then dropped back beyond the area influenced by the magnet. As a result, changes in concentrations at Outputs I and II were observed, which could be taken as a measure of the degree of mixing (see Figure 2).

To verify that the flow in the model setup remained laminar under the given conditions, we calculated the Reynolds numbers (Re) for different flow rates of water at 20 °C passing through a rectangular channel. The Reynolds number is a dimensionless quantity used to predict flow patterns in different fluid flow situations. Laminar flow typically occurs when the Re is below 2000, while higher values indicate a transition to turbulent flow.

The Reynolds number was calculated using the following equation:

(1) Re=ρ⋅ν⋅Dhμ

where

*ρ* is the density of water at 20 °C (998.2 kg/m^3^);

*v* is the average flow velocity (m/s);

*D*_ℎ_ is the hydraulic diameter of the channel (m);

*μ* is the dynamic viscosity of water at 20 °C (1.002 × 10^−3^ Pa·s).

For the calculation, the following parameters of the channel were assumed: The channel had a rectangular cross-section with dimensions of 2 mm × 5 mm. Hydraulic diameter *D*_ℎ_ for a rectangular channel was calculated as follows:

(2) $$D_{h}=\frac{2\cdot \left(w\cdot h\right)}{w+h}$$

where

w is the channel width (2 mm);

h is the channel height (5 mm).

The flow rates used in the calculations ranged from 1 mL/h to 50 mL/h, converted into m^3^/s for consistency in the units.

Table 4 displays the calculated Reynolds numbers for various flow rates; all the values fall well below 2000, confirming that the flow remains laminar under these conditions. This was essential for our study, as laminar flow provides the clear, predictable behavior necessary for observing the effects of a magnetic field on fluid mixing.

### 3.1. Spectrophotometric Analysis

**Figure 3:**
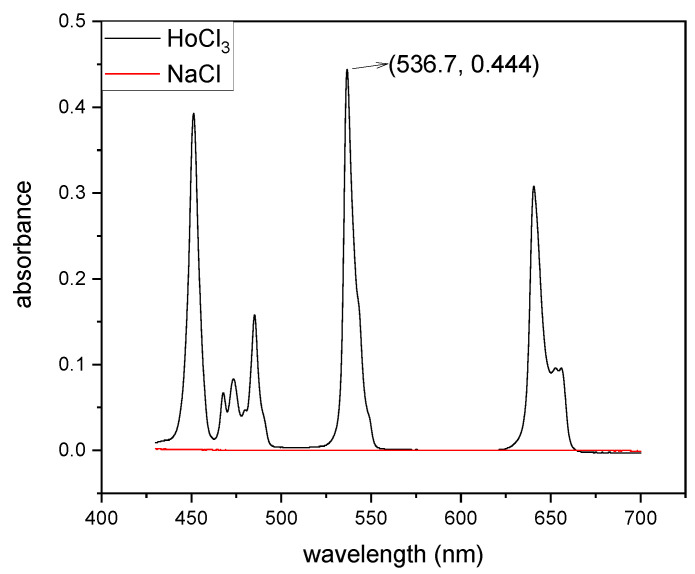
UV–vis spectra of the HoCl_3_ and NaCl solution.

The progress of mixing was monitored spectrophotometrically. The figure below (see Figure 3) presents the UV–vis spectra of a 0.1 M HoCl_3_ solution in 0.1 M HCl and NaCl. As seen in the spectra, the solution containing Ho^3+^ ions was colored, with the most intense peak observed at 536.7 nm. This peak was used as the primary indicator in monitoring the progress of mixing, as it provided a reliable and specific signal corresponding to the concentration of Ho^3+^ ions.

It is noteworthy that HCl did not exhibit any absorption bands in this spectral range, which ensured that the measurements of Ho^3+^ ions were not affected by interference from the solvent. The monitoring process was based on the Lambert–Beer law, which describes the relationship between absorbance and concentration in a linear manner. The law is expressed as follows:

(3) A=ε⋅l⋅c

where

*A* is the measured absorbance;

*ε* is the molar absorptivity (L·mol^−1^·cm^−1^);

*c* is the concentration of the absorbing species (mol·L^−1^);

*l* is the optical path length (cm).

Using this approach, the changes in the concentration of Ho^3+^ ions during the mixing process were quantitatively determined by monitoring the absorbance at 536.7 nm. This method ensured precise and reproducible tracking of the mixing progress in the microflow system. Additionally, the use of UV–vis spectroscopy provided real-time, non-invasive measurements, making it an ideal technique for the given experimental setup.

In our experimental setup, Output I (see Figure 1) was connected to a spectrophotometric flow cell, enabling the continuous monitoring of concentration changes. This configuration allowed for the real-time tracking of the mixing process by directly measuring the absorbance of the Ho^3+^ solution at the specified wavelength (536.7 nm) as it exited the microflow system. The use of a flow cell ensured that concentration changes could be recorded dynamically, providing high temporal resolution and eliminating the need for manual sampling during the experiment. This approach significantly enhanced the accuracy and efficiency of the spectrophotometric analysis.

The upper part (Output 1) concentration can be calculated as follows:

(4) $$C=\frac{A_{\lambda }}{\varepsilon_{\lambda }\cdot C}$$

where indices *λ* = 536.7 nm, and for the kinetic measurement of the flow through the cell, l = 5 mm, which is half of the optical length for measuring *A_λ_*_,0_ (the results are presented in Figure 1). Thus, we obtain the following:

(5) $$C_{Output\_I}=2\left(\frac{A_{\lambda }}{A_{\lambda ,0}}\right)\cdot C_{0}$$

and

(6) $$C_{Output\_II}=C_{0}-C_{Output\_I}=\left(1-\left(2\frac{A_{\lambda }}{A_{\lambda ,0}}\right)\right)\cdot C_{0}$$

The mixing efficiency equation was adapted from the literature [37] to fit our specific experimental setup involving two inlet channels and two outlet channels. The inlet concentrations of Ho(III) were set as follows: Inlet 1 contained a concentration of *C*_1_, while Inlet 2 had a concentration of *C*_2_. Due to monitoring constraints, measurements were conducted at only one outlet (Output 1). Consequently, the mixing efficiency equation required adjustments to account for mass balance.

Firstly, the ideal concentration for complete mixing (*C*∞) was determined assuming equal volumetric flows from both inlet channels, calculated as follows:

(7) $$C_{\infty }=\frac{C_{1}-C_{2}}{2}\cdot 100\%$$

The mixing efficiency (*η*_1_) at Output 1 was defined as follows:

(8) $$\eta_{1}=\left(1-\frac{\left|C_{Output1}-C\infty \right|}{\left|C_{Output1}^{0}-C\infty \right|}\;\right)\times 100\%$$

where

*C_Output_*_1_ represents the measured concentration at Output 1;

*C*∞ is the ideal fully mixed concentration;

$C_{Output1}^{0}$ is the theoretical concentration at Output 1 under conditions of no mixing, determined by the initial inlet concentrations and volumetric flow ratios.

Thus, by substituting the experimentally measured concentration at Output 1 into this equation, the mixing efficiency can be readily evaluated. For instance, a measured Ho(III) concentration of 0.045 M at Output 1 corresponds to a mixing efficiency of 90%.

### 3.2. PIV Analysis

By tracking the trajectories of fluorescent microparticles introduced into the flow, the velocity distribution within the microchannel was measured. This approach provided detailed insights into the flow dynamics, including the effects of the magnetic field on the formation of vortices and disturbances, which are critical for understanding the mixing process.

**Figure 4:**
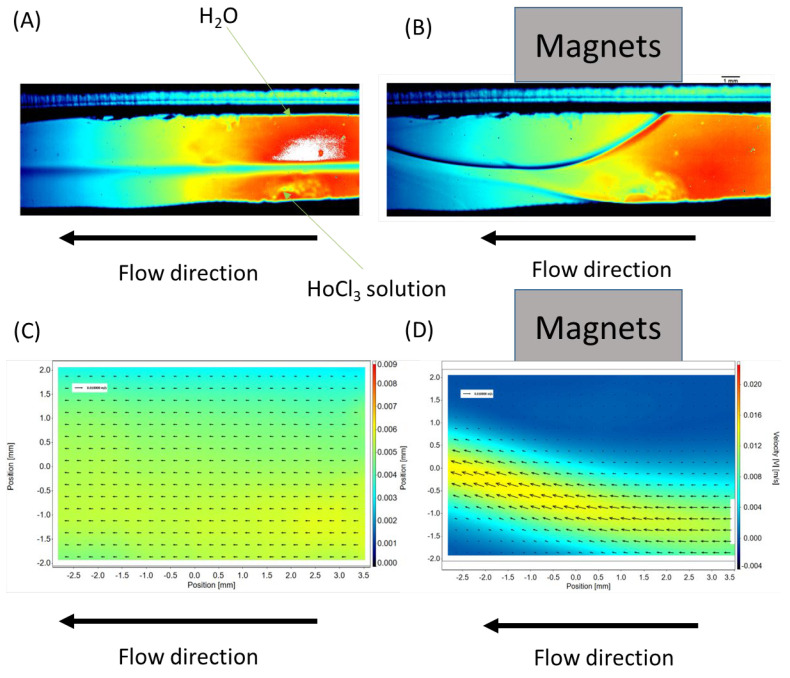
Examples of PIV images: (**A**) flow without a magnetic field, (**B**) flow after applying a magnetic field, (**C**) flow velocity distribution without a magnetic field, and (**D**) flow velocity distribution after applying a magnetic field.

The placement of a neodymium magnet near the surface of the microchannel (see Figure 4B) induces a significant change in the flow dynamics within the microreactor. The initially stable laminar flow becomes disrupted upon the application of the magnetic field. The solution containing magnetic ions, such as Ho^3+^, is attracted toward the neodymium magnet, creating a localized alteration in the flow pattern.

Simultaneously, UV–vis spectrophotometric analyses were conducted to monitor concentration changes in real time. In the absence of the magnet, no mixing was observed, and the concentration of Ho^3+^ ions at Output I remained negligible. Any small amounts of Ho^3+^ detected at Output I could be primarily attributed to diffusive mixing and minor imperfections in the separation system at the outlet. These effects were minimal and consistent with the expected behavior of a laminar flow system.

However, upon the application of the magnet, a sharp and dramatic disturbance in the concentration profile at Output I was recorded. The paramagnetic Ho^3+^ solution was strongly attracted to the region near the magnet, which caused significant alterations in the flow dynamics and resulted in noticeable deviations in the concentration at the outputs. This demonstrates the effectiveness of the magnetic field in enhancing mixing, even in a system where diffusion and laminar flow would otherwise dominate. These findings underscore the potential for magnetic fields to provide a powerful, non-mechanical method for promoting mixing in microflow systems.

Based on the data obtained using the PIV technique, the velocity distribution in the cross-section of the microchannel was determined. Figure 4C shows the velocity distribution within the channel in the absence of an external magnetic field. As observed, the flow is perfectly laminar, consistent with the Reynolds number values presented in Table 4.

Upon applying a neodymium magnet to the surface of the microreactor, a dramatic disturbance in the flow was observed. After approximately 5 s, the flow reached a pseudo-stabilized state. The velocity distribution under the influence of the magnetic field is shown in Figure 4D. It is important to note that the magnetic field not only caused a local change in flow direction, resulting in flow disturbances, but also significantly altered the flow velocity. The axes on the velocity distribution plots are expressed in mm/s and m/s.

In practice, the local flow velocity increased significantly (from a maximum velocity *V*_max_ of approximately 3 mm/s to a *V*_max_ of 0.004 m/s, or about 33%).

The presence of the “obstacle” directly near the magnet created local flow disturbances. These disturbances were characterized by the generation of vortices, which mimicked behavior typically associated with turbulent flow. This localized disruption may have enhanced the mixing efficiency by promoting chaotic advection, effectively increasing the interaction between phases. This phenomenon highlights the potential of magnetic field-induced flow perturbations to overcome the limitations of laminar flow in microfluidic systems and to significantly improve mixing performance in microreactors.

In the subsequent experiment, fluorescent particles were introduced exclusively into the stream containing Ho^3+^ ions. This approach allowed for the precise identification of what constituted “obstacles” within the channel—whether they were regions of water or areas rich in magnetic Ho^3+^) ions. By selectively tagging only the Ho^3+^-rich phase with fluorescent particles, it became possible to track its behavior under the influence of the magnetic field and its interaction with the surrounding non-magnetic water phase.

**Figure 5:**
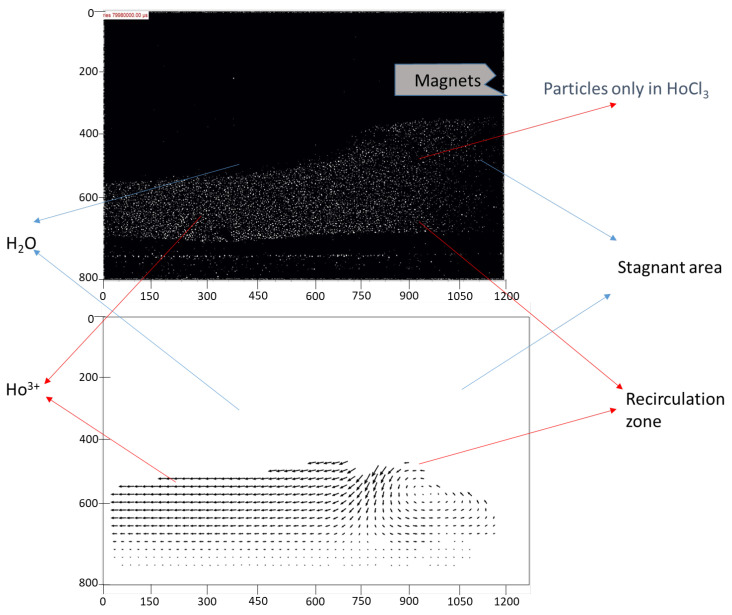
Velocity distribution of the Ho^3+^-rich phase when the magnets were applied.

The results of this analysis are presented in Figure 5. The fluorescence data clearly highlight the dynamic movement of the Ho^3+^-enriched phase and its tendency to be attracted toward the magnet. This provides direct evidence that the observed flow disturbances and obstacles within the channel were formed predominantly by the magnetic ion-rich phase. Moreover, these findings demonstrate the effectiveness of using fluorescent particles as tracers to visualize and quantify the behavior of specific phases in microfluidic systems.

This experimental setup not only validated the hypothesis regarding the localization of magnetic phases but also underscored the utility of advanced flow visualization techniques in studying complex interactions within microreactors. The results suggest that these obstacles, formed by the paramagnetic phase, play a crucial role in enhancing mixing by creating localized disruptions and promoting vortex formation.

**Figure 6:**
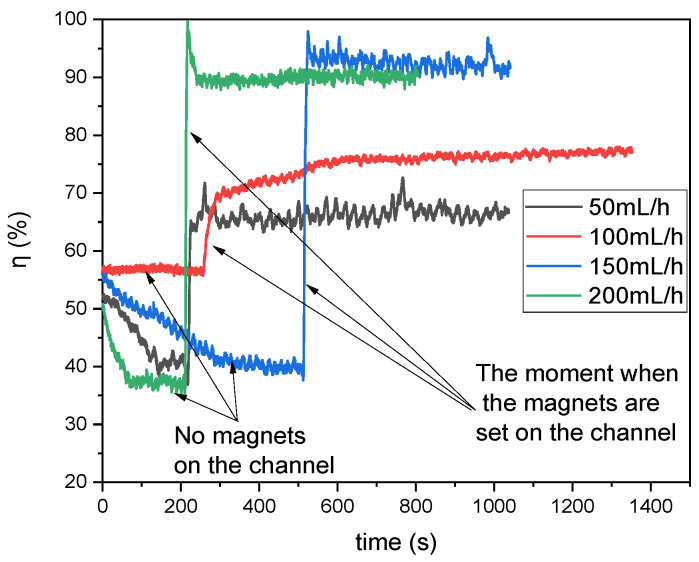
Degree of normal mixing with respect to the flow direction at different flow rates.

Figure 6 illustrates the recorded absorbance values converted into the degree of mixing (%) for various flow rates through the microreactor. The initial state should be considered as a transient phase (no magnets placed on top of the channel) observed immediately after the pumps were activated. Once the flow stabilized, a stack of five magnets was placed on the upper edge of the reactor. This resulted in an immediate change in the composition observed at Output 1. The moments in time when the magnets were applied on top of the channel are marked by arrows in Figure 6.

It can be observed that there is a clear correlation between flow rate and the degree of mixing. Specifically, lower flow rates generally result in lower mixing degrees. Conversely, higher flow rates enhance mixing efficiency; however, this improvement must be balanced against the significantly shorter residence time within the reactor. Thus, optimal flow conditions must be carefully selected to achieve effective mixing without compromising reaction completeness or efficiency. Moreover, specific fluctuations with relatively high amplitudes were observed in the mixing data. Initially, these fluctuations were thought to be noise caused by minor imperfections in the reactor’s surface or the pumps’ operational inconsistencies.

**Figure 7:**
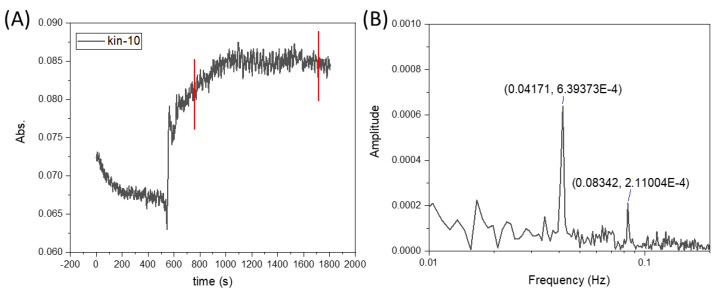
(**A**) Ho^3+^ concentration versus time with superimposed oscillations. (**B**) FFT analysis. The flow rate was 100 mL/h.

However, upon conducting a frequency analysis (see Figure 7), it became evident that these were not a result of random noise but rather systematically recurring fluctuations. This discovery suggests that the observed fluctuations are an inherent feature of the system dynamics under the influence of the magnetic field. Such behavior may be attributed to periodic disruptions in the flow caused by the interaction between the magnetic field and the paramagnetic phase, leading to localized vortex formation and unsteady mixing patterns.

These findings emphasize the complex nature of magnetic field-induced mixing in microreactors and the need for further investigation to understand the underlying mechanisms governing these systematic fluctuations.

The fluctuations observed in the experiments resulted from the interaction of the magnetic field with the flow of the liquid containing paramagnetic ions, which led to the periodic generation and decay of vortices near the magnetic obstacle. This phenomenon resembles the classical generation of von Kármán-type vortices, which are well known in fluid mechanics.

**Figure 8:**
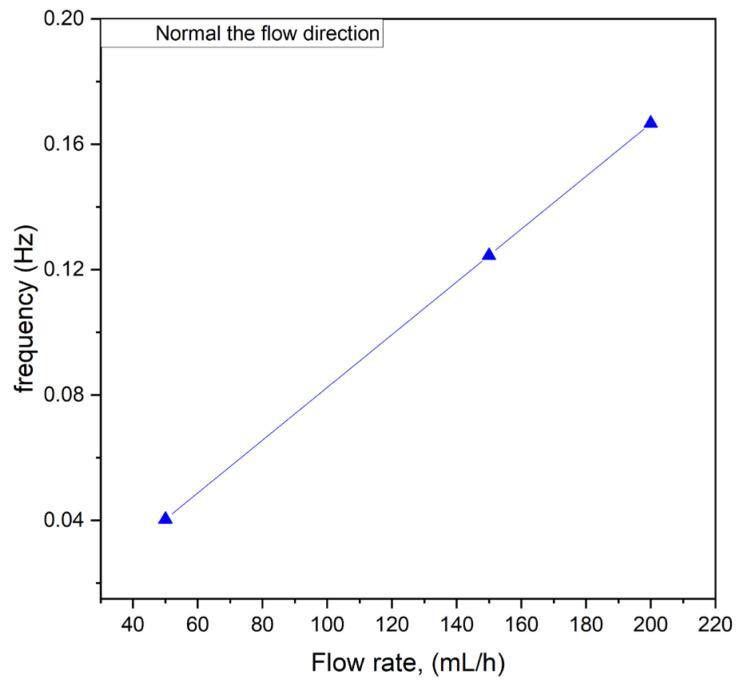
The influence of oscillation frequency vs. flow rate.

Additionally, studies were conducted to determine whether the flow rate influenced the frequency of these oscillations. The results are presented in Figure 8. As shown, there was a linear correlation between the flow rate and the frequency of the fluctuations within the tested flow rate range of 50 to 200 mL/h. This linear relationship suggests that the oscillatory behavior observed in the system was directly linked to the flow dynamics induced by the interaction between the magnetic field and the paramagnetic phase. At higher flow rates, the increased velocity of the paramagnetic solution likely enhanced the frequency of interactions with the magnetic field, resulting in more rapid and systematic fluctuations.

The observed correlation has significant implications for the design and operation of microfluidic systems employing magnetic field-induced mixing. By tuning the flow rate, it may be possible to control the frequency of oscillations and, consequently, influence the mixing efficiency. This finding underscores the importance of understanding flow rate as a critical parameter in optimizing microreactor performance and tailoring the system’s behavior to specific applications. Further investigation into the interplay between flow dynamics, oscillation frequency, and mixing outcomes could provide deeper insights into the mechanisms driving these phenomena.

### 3.3. COMSOL Multiphysics Simulations

**Figure 9:**
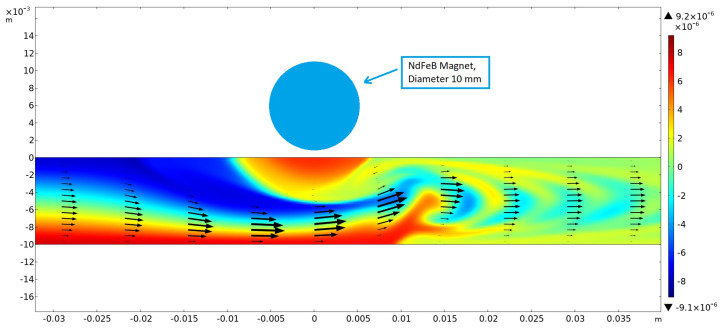
Vortex shedding enhanced the mixing downstream of a magnetic obstacle in the center upper part, as shown by a snapshot of the distribution of magnetic susceptibility. Re = 10; t = 500 s.

Numerical simulations were first performed in 2D without taking buoyancy into account. The magnetic field was generated using a cylindrical NdFeB magnet 10 mm in diameter, magnetized in the vertical direction and placed above the channel (height: 10 mm) at x = 0 with a gap of 1.25 mm. For this arrangement, the magnetic field strength below the center of the magnet amounted to 0.382 T at the upper channel wall. The results showed that, within a certain range of parameters describing the strength of the magnetic gradient force and the flow velocity, optimum mixing could be achieved (see Figure 8). This was caused by the creation of a magnetic obstacle next to the magnets, which triggered vortex shedding in the microchannel behind the obstacle that considerably enhanced mixing. The first investigations showed that the vortex-shedding frequency matched the frequency of the concentration oscillations measured and shown in Figure 7B and Figure 9.

**Figure 10:**
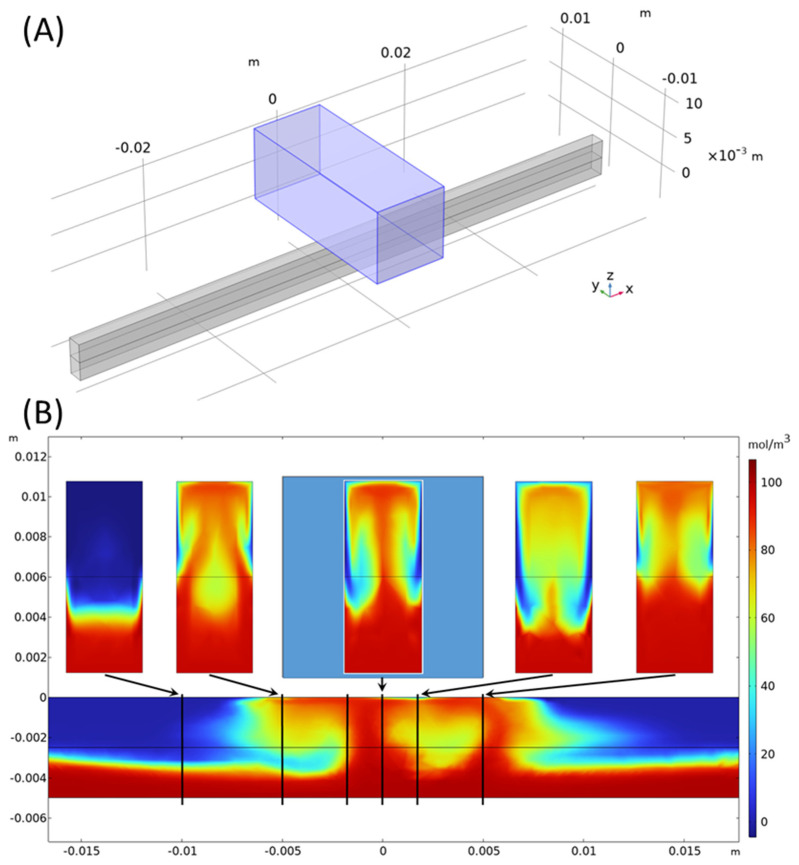
(**A**) Sketch of the geometry with a NdFeB quad magnet (magnetized in a z direction) placed above a channel with a rectangular cross-section. The flow is from left to right, and a horizontal center plane in the channel is marked in black. For further details, see text. (**B**) Three-dimensional simulation result at Re = 10 and t = 1 s. The concentration of the paramagnetic species in the streamwise vertical center-plane of the channel (at y = 0) is shown.

Further numerical simulations were performed in 3D for channels with a rectangular cross-section. It was found that the aspect ratio of the channel strongly influenced the onset of vortex shedding, as the sidewalls contributed a damping effect. This observation influenced the experimental investigation, leading to the selection of broader channels. However, transient 3D simulations are highly demanding, requiring significantly larger computational effort than 2D simulations. The spatial resolution and integration time currently required for obtaining accurate results in 3D require computation times that made extensive optimization studies impractical. Despite these challenges, the first results obtained from the 3D simulations were consistent with our experimental observations and closely matched the results of the 2D simulations. Figure 10 shows a 3D simulation result at Re = 10 and t = 1 s, where a 10 × 10 × 25 mm brick-shaped NdFeB magnet, magnetized in the vertical direction, was placed above a channel with a 2 × 5 mm rectangular cross-section with a gap of 1 mm. The magnetic field strength amounted to 0.393 T at the middle of the upper channel wall below the magnet center. As visible from the cross-section plot of the concentration at x = −10 mm, gravity in general tends to enhance the concentration in the lower channel half, thus acting against mixing. However, in the channel region below the magnet, paramagnetic liquid is lifted up towards the magnet, and enhanced mixing could be clearly observed. Further 3D simulations need to be performed in future work to study the effect in more detail and to perform broader investigations into parameter optimization.

The magnet above the channel is marked in light blue. At the x-positions of −10, −5, −2, 0, 2, and 5 mm, marked by vertical black lines, enlarged views of the concentration in the cross-section planes are shown above. The maximum concentration in the color bar is 110 mol/m^3^. For details, see Equation (7).

## 4. Discussion

The conducted studies demonstrated that the application of a magnetic field—specifically, a gradient magnetic field—has a strong effect on solutions containing magnetic substances. This phenomenon can be utilized to enhance mixing in microflow reactors. As shown by Wojtaszek et al. [20], a vast number of substances exhibit non-zero magnetic susceptibility, including organic compounds.

In our experiments, we used Ho^3+^ ions as a model system, aware that they possess a very high magnetic susceptibility [38]. However, in our setup, we employed relatively weak magnetic fields generated by permanent neodymium magnets. Despite the modest strength of the magnetic field, significant effects were observed, including oscillatory behavior in the system. These oscillations are, beyond any doubt, associated with the formation of magnetic obstacles in the channel volume due to the attraction of the magnetic phase towards the magnets.

The magnetic obstacle formed in this manner disrupted the laminar flow characteristic with respect to the low Reynolds number range in the microfluidic channel. The mixing process was caused by the vortices generated downstream, which considerably enhanced the interaction between the laminar layers. The results suggest that even weak magnetic fields, when paired with substances exhibiting high magnetic susceptibility, can induce substantial flow disturbances, offering a practical method for improving mixing in systems typically constrained by diffusion-dominated laminar flow.

The findings of this study not only confirm the potential of magnetic fields for enhancing microreactor performance but also highlight the broader applicability of the approach. Given that many substances, including various organic compounds, exhibit measurable magnetic susceptibility, this technique could be extended to a wide range of chemical and biological applications. Future work will focus on refining the system to achieve controlled mixing in environments with weaker magnetic substances and exploring the scalability of this method for industrial applications.

Furthermore, while Ho(III) ions were chosen in this study due to their high magnetic susceptibility, we acknowledge that their direct use in biological applications may be limited due to potential cytotoxicity. However, this technique can be extended to other paramagnetic substances that are biocompatible, such as iron-based complexes commonly used in MRI imaging. Additionally, the mixing system could be adapted to operate with external magnetic actuation, reducing direct contact between the magnetic substance and the biological sample, thus making it more suitable for sensitive applications.

Our method based on magnetically induced mixing stands out compared to techniques utilizing ultrasonic waves or active chaotic mixers in terms of energy efficiency, simplicity of design, and applicability in microfluidic systems.

The key differences include the following:

Energy efficiency: Our method relies on permanent magnets that do not require an external energy source. In contrast, ultrasonic or electrical methods may generate additional energy losses, affecting the overall efficiency of the system.

Degree of mixing: While ultrasonic and chaotic mixing can achieve high degrees of mixing, our method allows effective mixing under low Reynolds number conditions without the need for complex channel geometries. This makes it particularly useful in microfluidic applications where flow control is crucial.

Simplicity of design: Our approach eliminates the need for moving parts and additional energy sources, facilitating implementation in existing microfluidic systems. The simplicity of the design translates to lower production and maintenance costs, as well as increased system reliability.

An additional advantage of our method is the precise control of the mixing process. By using permanent magnets, it is possible to accurately control the intensity and direction of mixing, allowing for better adaptation of the process to specific application requirements. A further important aspect is minimizing the impact on the sample. Ultrasonic methods can cause undesirable effects, such as sample heating or the degradation of sensitive substances. Our method minimizes such risks, which is important in biochemical or medical applications. Moreover, the proposed method can be easily integrated with other technologies due to its simplicity and the lack of need for external power, thus opening up new possibilities in designing advanced analytical systems.

However, as microchannels become smaller, particularly below 100 µm in size, several challenges arise. Among them, the tracer particles used in micro-PIV must be small enough to accurately follow the fluid flow without altering it. In very narrow channels, even particles with diameters of a few micrometers can disrupt the flow, leading to inaccurate measurements. This is especially problematic when the particle size approaches the dimensions of the microchannel. Next, the spatial resolution of micro-PIV is constrained by optical diffraction limits and the size of the tracer particles. Achieving high-resolution measurements in sub-100 µm channels is challenging due to these limitations. The finite size of the measurement volume can lead to averaging effects, reducing the accuracy of velocity measurements in confined spaces. Additionally, at microscales, tracer particles are subject to Brownian motion, which introduces noise into the velocity measurements. This random motion becomes more significant as the particle size decreases, further complicating accurate flow characterization.

It might seem that a limitation of the method is the need to use substances with sufficiently high magnetic susceptibility to induce noticeable mixing effects. Although the method may be significantly less efficient for diamagnetic or nonmagnetic substances, reasonable efficiency could be maintained through further improvements in the magnets’ arrangement to generate stronger magnetic gradients with large field strengths. Additionally, our yet-unpublished results show that even the weaker magnetic properties of water are sufficient for corresponding fluctuations in the flow to be generated (when mixing water with, for example, an ethanol-based solution).

Compared to other active mixing methods, such as ultrasound and electric oscillating fields, our approach is characterized by low operating costs, as it does not require a continuous supply of energy. The differences in energy costs between the magnetic method and other active mixing methods will depend on the channel geometry and the flow scale. To more precisely determine the energy cost resulting from pressure drops, we plan to conduct experiments using high-sensitivity pressure sensors. This will allow us to precisely determine the effect of the magnetic field on the required pumping pressure and allow for a quantitative comparison of our method with other active mixing techniques.

## 5. Conclusions

At the outset, it should be emphasized that the distinction between active and passive mixing is not entirely clear. Some authors define active mixing as any approach that introduces additional energy into the system to increase mixing intensity. In our case, we employed permanent magnets, which generated a magnetic field that acted on the flow and modified it, thereby causing an increased pressure drop. Thus, additional energy is required for effective mixing, which is ultimately provided by the pumping system. Consequently, it must be acknowledged that the use of such a mixing method will lead to higher flow resistance and thus potentially increased energy consumption from the pumps.

Despite this consideration, the observed phenomenon offers significant practical benefits. Moreover, the mixing process can be easily intensified by strategically placing multiple magnets along the length of the microreactor. This is particularly important in pharmaceutical applications, where the high cost of reagents and the limited availability of certain materials, such as human-derived biological samples, demand efficient and cost-effective methods. Microreactors enable experiments to be conducted with minimal reagent volumes, often in the picoliter range.

A persistent challenge in microfluidic systems is the dominance of laminar flow, where mixing relies primarily on diffusion. Numerous strategies have been explored to enhance mixing under such conditions, but most involve moving parts or complex channel geometries, complicating both the design and operation of the reactor. The method proposed in this study overcomes these constraints. It relies solely on magnetic fields, eliminating the need for moving components, and can be applied to virtually any type of reactor that does not absorb magnetic field lines. As a result, the fabrication of the microreactor remains straightforward, while the mixing system operates externally, independent of the reactor’s structure. This simplicity and flexibility render the proposed approach particularly suitable in scenarios where maintaining low reagent consumption, operational ease, and cost efficiency are paramount.

The best example is our other study on a single-cell drug-screening platform, where we analyzed the response of single cells to drugs. In such experiments, we used a small number of cells that had to be placed in a microenvironment with a precisely controlled and homogeneous concentration of the active substance. We already know from experience that the use of ultrasound in such conditions leads to mechanical destruction of the cells, which makes this method unsuitable for such applications. Our magnetic mixing method eliminates this problem because it does not introduce excessive shear forces or rapid hydrodynamic disturbances into the system. This is a key advantage in biomedical applications, where the delicate manipulation of cells and biomolecules is a must.

By optimizing the placement and strength of the magnets, this system could provide benefits in a wide range of applications, from pharmaceuticals to biological research, ensuring rapid and efficient mixing across numerous microfluidic setups. Although this method does lead to increased flow resistance due to the induced disturbances, its advantages in terms of simplified construction, adaptable implementation, and compatibility with ultra-small reagent volumes underscore its exceptional potential for various scientific and industrial fields.

## Figures and Tables

**Table 1:** Analysis of mixing methods and their advantages and challenges.

Mixing Method	Mechanism	Advantages	Challenges
Acoustic Mixing	SAW-driven fluid jets	High controllability, rapid mixing [19]	Energy inefficiency in some configurations [20]
Chaotic Mixing	Geometrical channel designs	Efficient at high Re, no external energy [21]	Complex designs, less effective at low Re [22,23]
Ultrasonic Mixing	Acoustic body force	Rapid, efficient mixing	Direction-dependent performance [24]
Magnetic Mixing	Magnetic obstacles, localized vortices	Effective for specific fluids, precise control	Requires magnetic properties in fluids [25]

**Table 2:** Magnetic susceptibility values (χ) of selected water-soluble substances expressed in units of 10^−6^ cm^3^/mol.

Substance	Magnetic Susceptibility, 10^−^⁶ cm³/mol
Water	−9.05
Holmium chloride (HoCl_3_)	2830
Iron sulfate (FeSO_4_)	220
Copper sulfate (CuSO_4_)	16
Ethanol	−5.2
Acetone	−6.7
Sodium chloride (NaCl)	−12

**Table 3:** Correlation between the number of magnets and the strength of the magnetic field at the center of the large bottom surface.

Number of Magnets	Magnetic Field Strength, T
1	0.3016
2	0.4338
3	0.4875
4	0.5065
5	0.5177
6	0.5331
7	0.5336
8	0.5506
9	0.5546
10	0.5555
11	0.5566
12	0.5517
13	0.5516
14	0.5555
15	0.5481

**Table 4:** Calculated Reynolds numbers for different flow rates in a rectangular channel to confirm laminar flow.

Flow Rate (mL/h)	Reynolds Number
50	3.953
100	7.906
150	11.859
200	15.812

## Data Availability

The original contributions presented in the study are included in the article, further inquiries can be directed to the corresponding author.
